# The Long Pentraxin PTX3 Promotes Fibrocyte Differentiation

**DOI:** 10.1371/journal.pone.0119709

**Published:** 2015-03-16

**Authors:** Darrell Pilling, Nehemiah Cox, Varsha Vakil, J. Sjef Verbeek, Richard H. Gomer

**Affiliations:** 1 Department of Biology, Texas A&M University, College Station, Texas, United States of America; 2 Department of Biochemistry and Cell Biology, Rice University, Houston, Texas, United States of America; 3 Department of Human Genetics, Leiden University Medical Center, Leiden, The Netherlands; University Medical Center Freiburg, GERMANY

## Abstract

Monocyte-derived, fibroblast-like cells called fibrocytes are associated with fibrotic lesions. The plasma protein serum amyloid P component (SAP; also known as pentraxin-2, PTX2) inhibits fibrocyte differentiation *in vitro*, and injections of SAP inhibit fibrosis *in vivo*. SAP is a member of the pentraxin family of proteins that includes C-reactive protein (CRP; PTX1) and pentraxin-3 (PTX3). All three pentraxins are associated with fibrosis, but only SAP and CRP have been studied for their effects on fibrocyte differentiation. We find that compared to SAP and CRP, PTX3 promotes human and murine fibrocyte differentiation. The effect of PTX3 is dependent on FcγRI. In competition studies, the fibrocyte-inhibitory activity of SAP is dominant over PTX3. Binding competition studies indicate that SAP and PTX3 bind human FcγRI at different sites. In murine models of lung fibrosis, PTX3 is present in fibrotic areas, and the PTX3 distribution is associated with collagen deposition. In lung tissue from pulmonary fibrosis patients, PTX3 has a widespread distribution, both in unaffected tissue and in fibrotic lesions, whereas SAP is restricted to areas adjacent to vessels, and absent from fibrotic areas. These data suggest that the relative levels of SAP and PTX3 present at sites of fibrosis may have a significant effect on the ability of monocytes to differentiate into fibrocytes.

## Introduction

During fibrosis, some monocytes leave the circulation, enter the tissue, and differentiate into fibroblast-like cells called fibrocytes [1–3]. Fibrocytes express markers of both hematopoietic cells (CD34, CD45, FcγR, LSP-1, MHC class II) and stromal cells (collagens and matrix metalloproteases) [2–6]. Fibrocytes are found in lesions associated with fibrotic diseases such as pulmonary fibrosis, congestive heart failure, cirrhosis of the liver, and nephrogenic systemic fibrosis [3,7–11]. In healthy tissues, there are very few fibrocytes [3]. Fibrocyte differentiation is regulated by several factors including the plasma protein Serum Amyloid P component (SAP; PTX2) [3,5,12,13].

SAP is a pentameric protein that belongs to the pentraxin family of evolutionarily conserved proteins [14]. There are three main systemic pentraxins in mammals: SAP, C-reactive protein (CRP), and the long pentraxin PTX3 [15]. SAP, CRP, and PTX3 all have regulatory roles in the immune system [16]. Pentraxins bind with different affinities to adhesion molecules, pathogens, and apoptotic cells, leading to complement activation, phagocytosis, and cytokine production [15]. In contrast to SAP and CRP, which are produced by hepatocytes, PTX3 is produced by macrophages, neutrophils, endothelial cells, epithelial cells, and fibroblasts [15]. In healthy humans, the plasma levels of CRP and PTX3 are low (< 2 μg/ml and < 25 ng/ml respectively), whereas during inflammation CRP and PTX3 levels may rise to 500 μg/ml and 1000 ng/ml respectively [17–19]. In humans, plasma SAP levels are 20–60 μg/ml, and are not affected by inflammation [20].

Injections of SAP inhibit fibrosis in mouse models of pulmonary fibrosis, ischemic cardiac fibrosis, and renal fibrosis [8,21–24], and in a phase 1b clinical trial, SAP injections appeared to improve lung function in pulmonary fibrosis patients [25]. In mice, overexpression of CRP strongly potentiates inflammation and fibrosis [26–29]. PTX3 is associated with inflammation in humans, but in mice appears to be pro-inflammatory in some models and limits inflammation in other models [30,31].

SAP inhibits fibrocyte differentiation partly through a group of receptors called Fcγ receptors [22,32–35]. In humans, there are four activating FcγR: FcγRI, FcγRIIA, FcγRIIIA, and FcγRIIIB [36,37]. In mice, there are three activating Fcγ receptors: FcγRI, FcγRIII, and FcγRIV [36,38]. The activating receptors require an accessory common gamma chain (FcRγ), for plasma membrane localization and initiating a signaling cascade [39]. Both humans and mice have an inhibitory receptor, FcγRIIb, that triggers an inhibitory signaling pathway to help modulate the immune response [40]. We have previously found that FcγRI and FcRγ are responsible for the effect of SAP on fibrocyte differentiation in both humans and mice [32]. In addition, the inhibition of cardiac or renal fibrosis in mice by injections of SAP is also dependent on FcRγ [22,33].

Although all three main pentraxins, and fibrocytes, are associated with fibrosis, only SAP and CRP have been studied for their effects on fibrocyte differentiation. In this report, we find that PTX3 promotes fibrocyte differentiation. The effect of PTX3 is dependent on FcγRI, but in competition studies, the fibrocyte-inhibitory activity of SAP is dominant over PTX3. These data suggest that the relative levels of SAP and PTX3 present at sites of fibrosis may have a significant effect on the ability of monocytes to differentiate into fibrocytes.

## Materials and Methods

### Human PBMC, leukocyte, and monocyte isolation, and cell culture

Human peripheral blood was collected into heparin tubes (BD Bioscience, San Jose, CA) from healthy adult volunteers who gave written consent and with specific approval from the Texas A&M University human subjects Institutional Review Board. Peripheral blood mononuclear cells (PBMC) were isolated from the blood using Ficoll-Paque Plus (GE Healthcare Biosciences, Piscataway, NJ), as described previously [5,12,41,42]. CD14+CD16- monocytes were enriched from PBMC using EasySep monocyte enrichment kits, following the manufacturer’s instructions (StemCell Technologies, Vancouver, Canada). Monocytes were checked for enrichment by flow cytometry in comparison to the un-enriched PBMC population, as described previously [12,13,41,43,44]. Monocyte preparations were 93.6% ± 2.5 (mean ± SEM, n = 3) CD14 positive and < 0.5% positive for T cells, B cells, and NK cells.

Human leukocytes were isolated from blood using Lympholyte-poly (Cedarlane Laboratories, Hornby, BC) following the manufacturer’s protocol [45]. HEK293 cells (Life Technologies, Grand Island, NY) were cultured in Freestyle (Life Technologies) medium following the manufacturer’s protocol. K562 cells (ATCC, Manassas, VA) were grown in RPMI 1640 with 10% FBS (Hyclone-GE Healthcare Life Sciences, South Logan, UT). Human dermal (PromoCell, Heidelberg, Germany) and MRC5 lung (Sigma, St Louis, MO) fibroblasts were cultured in DMEM with 10% FCS. Human lung bronchial epithelial cells (PromoCell) were incubated in epithelial cell growth medium (PromoCell) following the manufacturer’s protocol.

### Cell fractionation of murine spleen cells

All experimental protocols were approved by the local ethical committees and performed in accordance with national guidelines and regulations. This study was carried out in strict accordance with the recommendations in the Guide for the Care and Use of Laboratory Animals of the National Institutes of Health. The protocol was approved by the Texas A&M University Animal Use and Care Committee (TAMU AUP 2009-262 and 2013-272). Mice maintained at Leiden University Medical Center were monitored according to the rules of the Federation of European Laboratory Animal Science Associations (FELASA) and with approval of the Dierexperimentencommissie Academisch Ziekenhuis Leiden (Animal Experiments Committee Academic Hospital Leiden). Procedures at Leiden were performed under ketamine/atropine/xylazine anesthesia, and euthanasia at both institutions was achieved by CO_2_ asphyxiation followed by cervical dislocation. All efforts were made to minimize suffering. Spleen cells from 4–6 week male C57BL/6J mice (Jackson Laboratories, Bar Harbor, ME), and from FcγRI, FcγRIIb, FcγRIII, and FcγRI/IIb/III/IV knockout mice on the C57BL/6 background generated at Leiden University Medical Center [46–48], were used in this study. Mouse spleen cells were isolated as described previously [32,49–51].

### Fibrocyte differentiation assay

Human PBMC were cultured in FibroLife (LifeLine Cell Technology, Walkersville, MD) defined serum-free medium (SFM), as described previously [6,42], in the presence or absence of purified human SAP (EMD Millipore, Billerica, MA), purified human CRP (EMD Millipore or Fitzgerald Industries, Acton, MA), or mammalian NSO cell-derived recombinant human PTX3 (R&D Systems, Minneapolis, MN). As commercial SAP and CRP preparations contain 0.1% azide, we buffer-exchange the SAP into 20 mM sodium phosphate, pH 7.4 and the CRP into 150 mM NaCl, 20 mM Tris, 2 mM CaCl_2,_ pH 7.4, as described previously [5,6,12,49]. After 5 days, plates were air-dried, fixed with methanol, and stained with eosin and methylene blue, as described previously [5,12,32,41,42,49]. Fibrocytes were identified and counted based on the following criteria: an adherent cell with elongated spindle-shaped morphology and an oval nucleus, in five different 900 μm-diameter fields of view per well as described previously [5,32,41,42,49].

Murine spleen cells were cultured in FibroLife (Lifeline Cell Technology, Frederick, MD) serum-free medium (SFM) including 50 ng/ml murine IL-13 and 25 ng/ml murine M-CSF (PeproTech, Rocky Hill, NJ), as described previously [32,42,49]. On day 3 of the incubation, wells were supplemented with IL-13 and M-CSF, as described previously [32,49]. After 5 days, plates were dried and fibrocytes counted as described above.

### Bleomycin-induced lung inflammation

This study was carried out in strict accordance with the recommendations in the Guide for the Care and Use of Laboratory Animals of the National Institutes of Health. The protocol was approved by the Texas A&M University Animal Use and Care Committee (TAMU AUP #2009-0265 and #2013-0007). All procedures were performed under anesthesia, and all efforts were made to minimize suffering. 4–6 week old C57BL/6 mice (Jackson Laboratory, Bar Harbor, ME) were treated with an oropharyngeal aspiration of 50 μl of 3 U/kg bleomycin (EMD Millipore, Billerica, MA) solution in 0.9% saline or saline alone, as described previously [50,52]. Mice were euthanized at 21 days after bleomycin aspiration, and the lungs were removed and inflated with pre-warmed optimal cutting temperature (OCT) compound (VWR, Radnor, PA), embedded in OCT, frozen on dry ice, and stored at −80°C as described previously [21,50,52].

### Immunohistochemistry and flow cytometry

PBMC, fibroblasts, or lung epithelial cells were cultured for 5 days on eight-well glass microscope slides (Millipore), and then fixed and stained as described previously [5,6]. The cells were stained with mouse monoclonal antibodies to CD13 (WM15, BD-Biosciences, San Jose, CA), CD14 (HCD14, BioLegend, San Diego, CA), CD34 (QBend10, Beckman Coulter, Brea CA), CD45 (H130, BioLegend), CD90 (5E10, BioLegend), CXCR4 (44716, R&D Systems), S100A9 (MAC387, AbD Serotec, Raleigh, NC), MHC class II (L243, BioLegend), PM-2K (Abcam, Cambridge, MA), fibronectin (EP5, GeneTex, Irvine, CA), or rabbit polyclonal antibodies to collagen-I (Abcam), collagen VI (Novus Biologicals, Littleton, CO), PTX3 (GeneTex), SAP (EP1018Y, Abcam), or EpCAM (ab71916, Abcam). Isotype-matched irrelevant mouse monoclonal antibodies (BioLegend) or control rabbit polyclonal antibodies (anti-chicken IgY, Bethyl Laboratories, Montgomery, TX), were used as controls. Secondary F(ab’)_2_ biotin-conjugated donkey anti-mouse or F(ab’)_2_ biotin-conjugated donkey anti-rabbit antibodies were from Jackson ImmunoResearch (West Grove, PA). Staining was revealed with streptavidin-alkaline phosphatase (Invitrogen, Grand Island, NY) and Vector Red Alkaline Phosphatase Kit (Vector Laboratories, Burlingame, CA) following the manufacturers’ instructions, and slides were then counterstained with hematoxylin [5,6]. Human lung tissue sections were obtained from the National Heart Lung and Blood Institute-sponsored Lung Tissue Research Consortium (LTRC, Bethesda, MD). Murine and human lung sections were prepared and incubated with rabbit antibodies to PTX3 (GeneTex) or SAP (Abcam), as described previously [6,50]. Isotype-matched rabbit anti-chicken IgY antibodies (Bethyl) were used as controls. Images were analyzed with ImageJ (NIH, Bethesda, MD), as described previously [6,50]. For flow cytometry, PBMC were cultured for 5 days in the presence or absence of 1 μg/ml PTX3. Non-adherent cells were then removed, the adherent cells detached by treatment with trypsin-EDTA, and cells were stained for intracellular collagen-VI, as described previously [49].

### PTX3 and SAP binding assays and receptor expression

Human FCGR1A, Fc common γ-chain (FCER1G), and FCGRIIIB cDNAs (PSI:Biology-materials repository, Tempe, AZ) [53] were ligated into the pCMV6-AC-His vector (OriGene, Rockville, MD) and then transfected into HEK293 cells using a 4D-Nucleofector electroporation system (Lonza, Cologne, Germany) following the manufacturer’s protocol. HEK293 cells expressing human FcγRI or FcγRIIIB and K562 cells (which express human FcγRIIA) [54], were used to measure the affinity of PTX3 and SAP for FcγR, as described previously [45]. PTX3 and SAP were labeled using Alexa Fluor 647-NHS (Life Technologies) following the manufacturer’s protocol. The binding of fluorescently labeled PTX3 or SAP was then measured as previously described using an Accuri C6 flow cytometer [45]. K562, HEK293, FcγRI+ HEK293, and FcγRIIIB+ HEK293 cells were stained for FcγRI (clone 10.1; BioLegend), FcγRII (clone FUN-2; BioLegend), and FcγRIII (clone 3G8; BioLegend) to determine the expression of the indicated receptor by flow cytometry [6,45]. Leukocytes stained for CD3 (BioLegend), CD14 (BioLegend), CD16 (BioLegend), CD19 (BioLegend), CD45 (BioLegend), FcγRI, FcγRII, and FcγRIII were assayed by flow cytometry to determine the presence of different immune cell populations as previously described [6,45]. For competition experiments, leukocytes were incubated for 30 minutes at 4°C in PBS with 2% IgG-free BSA (Jackson ImmunoResearch), containing 1 μg/ml unlabeled PTX3, SAP, or human IgG1 Fc region (Fc block, BD Biosciences). After 30 minutes, an equal volume of PBS-BSA containing 2 μg/ml (1 μg/ml final) fluorescently labelled PTX3 or SAP was added and the cells were incubated for an additional 30 minutes. For competition experiments involving FcγRI+ HEK293 cells, cells were incubated for 30 minutes at 4°C in PBS with 2% IgG-free BSA, containing the indicated concentrations of unlabeled PTX3 or SAP, and 0.5 μg/ml fluorescently labelled PTX3 or SAP. Cells were then washed twice in ice cold PBS, resuspended in PBS-BSA, and assayed by flow cytometry.

### Statistical analysis

Statistical analysis was performed using Prism (GraphPad Software, San Diego, CA). Statistical significance between two groups was determined by t test, or between multiple groups using analysis of variance (ANOVA), with Tukey’s post-test. Significance was defined as p < 0.05. Data were fit to the appropriate model of binding as determined by F-tests.

## Results

### PTX3 potentiates human fibrocyte differentiation

**Fig 1:**
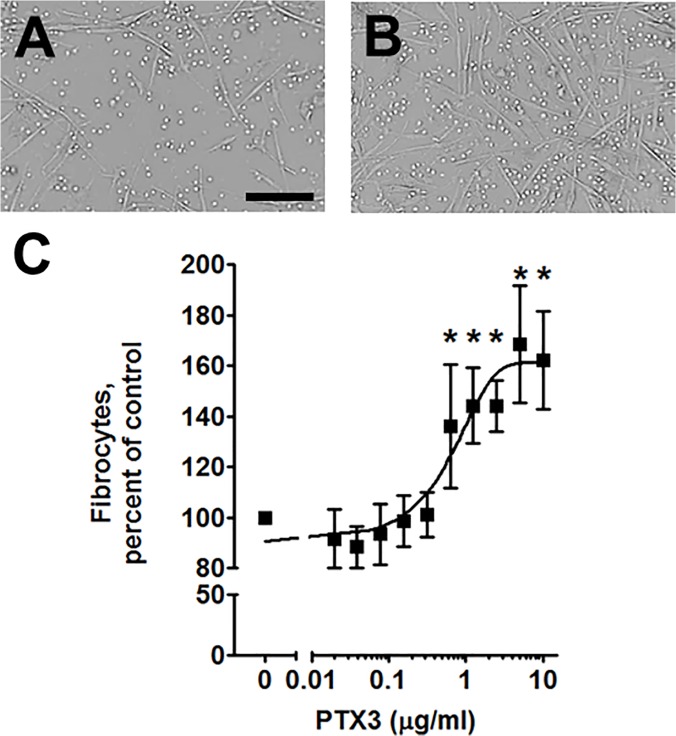
PTX3 promotes fibrocyte differentiation. Human PBMC were incubated for 5 days in the absence A) or presence B) of 1 μg/ml PTX3. Bar is 50 μm. C) The effect of the indicated concentrations of PTX3 on fibrocyte numbers. For each donor, numbers were normalized to the no-PTX3 control. Values are mean ± SEM (n = 4). *p <0.05 (t-test). Line is a fit to a sigmoidal dose response curve.

SAP and CRP bind to multiple FcγR on monocytes, but SAP inhibits monocyte to fibrocyte differentiation, whereas CRP does not [5,34]. PTX3 also binds to FcγR [34,55], and we thus examined whether PTX3 affects fibrocyte differentiation. Human PBMC were cultured for 5 days with or without PTX3. In the absence of added PTX3, we observed 420 to 1,600 fibrocytes per 10^5^ PBMCs from the different donors, similar to what we have previously observed [5,12,56]. Because of this variability, for each donor, fibrocyte numbers were normalized to pentraxin-free controls. For all donors, 0.625 μg/ml and above PTX3 significantly increased the number of spindle-shaped cells (Fig. 1), with an EC50 of 0.59 ± 0.20 μg/ml (mean ± SEM, n = 5).

**Fig 2:**
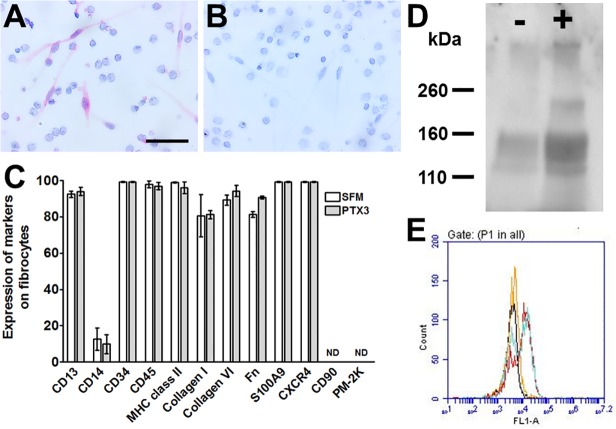
Effect of PTX3 on morphology, collagen production, and receptor expression. After 5 days incubation with 1 μg/ml PTX3, PBMC were air-dried, fixed, and stained with A) anti-collagen-I antibodies or B) control antibodies. Positive staining was identified by red staining, and nuclei are counterstained blue. Bar is 50 μm. C After 5 days, PBMC were air-dried, fixed, and stained with antibodies. Following immunocytochemical staining, at least 100 elongated cells with oval nuclei were examined from at least 10 randomly selected fields, and the percentage of positive cells is expressed as the mean ± SEM (n = 3–5 separate donors). ND – none detected. D) Supernatants from PBMC incubated in the absence (-) or presence (+) of 1 μg/ml PTX3 were assessed by western blotting, using anti-collagen I-V antibodies. Blot is representative of three separate experiments. E) After 5 days, adherent cells (macrophages and fibrocytes) were stained with collagen-VI (teal line SFM, red +PTX3) or control IgY (black SFM, orange +PTX3) antibodies. The data are representative of three separate experiments.

To determine whether the spindle-shaped cells were fibrocytes, and if PTX3 alters the phenotype of fibrocytes, we used immunocytochemistry to stain cells for fibrocyte markers (Fig. 2). In the presence or absence of PTX3, as we previously observed for fibrocytes [6], the elongated cells were positive for markers expressed by fibrocytes including CD13, CD34, CD45, MHC class II, CXCR4, S100A9, and collagen-I, collagen VI, and fibronectin (Fig. 2C). As we observed previously [6], all monocyte-derived cells cultured in SFM had low expression of the monocyte marker CD14, and fibrocytes showed no observable staining for the fibroblast marker CD90, or the tissue macrophage marker PM-2K. These data indicate that the elongated cells are fibrocytes (Fig. 2C). By immunocytochemistry, we did not observe any obvious effect of PTX3 on the staining intensity of the fibrocyte markers, indicating that PTX3 does not appear to alter the characteristics of fibrocytes. Fibrocytes secrete collagen [57], and we observed that 1 μg/ ml PTX3 caused an increase in the levels of soluble collagens (Fig. 2D), but not the levels of collagen-VI within individual cells (Fig. 2E). These data indicate that levels of human PTX3 similar to what can be observed in human plasma during inflammation promote human fibrocyte differentiation, but do not appear to alter fibrocyte characteristics.

### PTX3 acts directly on monocytes

**Fig 3:**
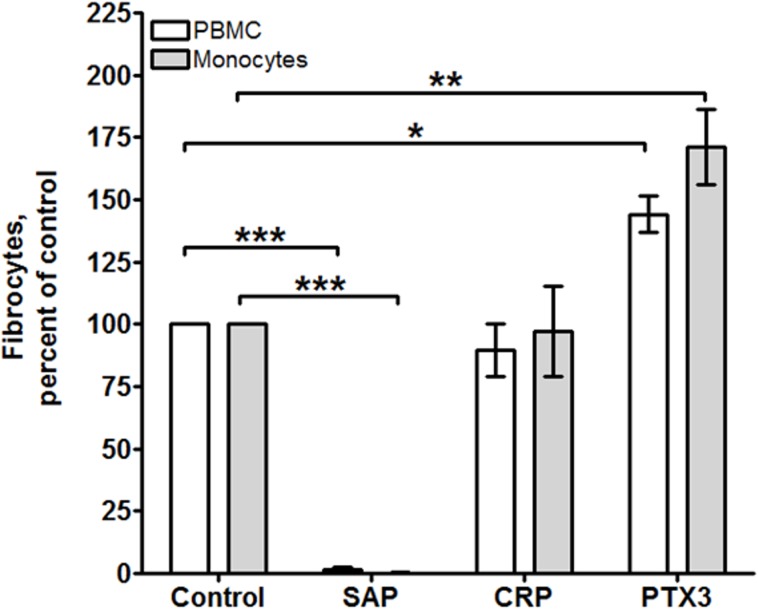
PTX3 acts directly on CD14+ CD16- (FcγRIIIA) monocytes. PBMC and isolated CD14+ CD16- monocytes were incubated for 5 days in the presence or absence of 1 μg/ml SAP, CRP, or PTX3. After 5 days, cells were air-dried, fixed, and stained, and the number of fibrocytes was counted. Values are mean ± SEM (n = 3). *p <0.05; **p <0.01; *** <0.001 (ANOVA).

To determine whether the potentiation of fibrocyte differentiation by PTX3 is a direct effect on monocytes, or due to an indirect effect on the T cells, B cells, or NK cells present within the PBMC preparation, we incubated purified CD14+ CD16- monocytes with PTX3 (Fig. 3). As previously observed, SAP inhibited, but CRP had no effect, on monocyte to fibrocyte differentiation [5,12]. For all donors, 1 μg/ml PTX3 significantly potentiated fibrocyte differentiation (Fig. 3). These data suggest that PTX3 acts directly on monocytes to potentiate fibrocyte differentiation.

### SAP inhibits PTX3-induced fibrocyte differentiation

**Fig 4:**
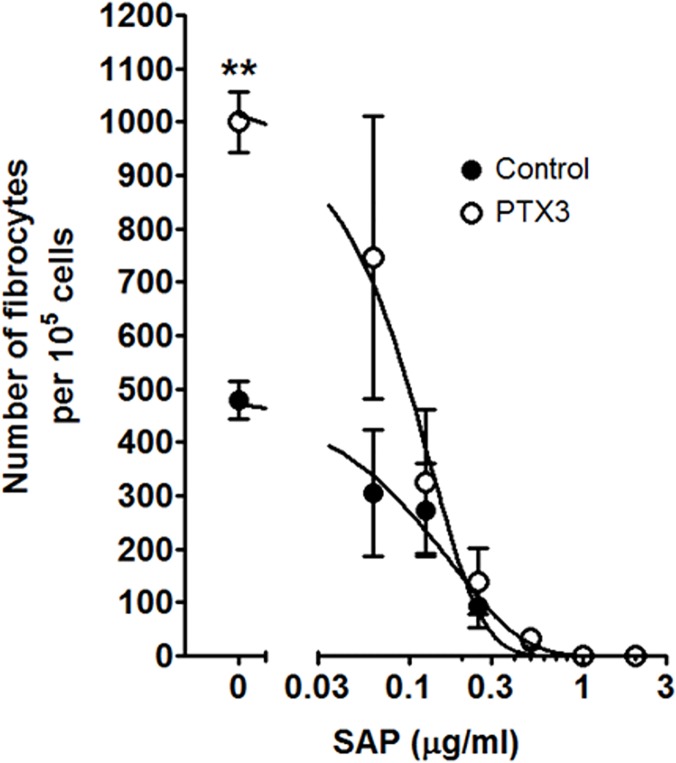
SAP inhibits fibrocyte differentiation in the presence of PTX3. PBMC were incubated with increasing concentrations of SAP in the presence or absence of 1 μg/ml PTX3. After 5 days, cells were air-dried, fixed, and stained, and the number of fibrocytes was counted. Values are mean ± SEM (n = 3). **p <0.01 (t-test). Lines are fits to sigmoidal dose response curves with variable Hill coefficients.

As fibrotic environments rarely, if ever, contain just one type of pentraxin, we examined how SAP and PTX3 might compete with each other. PBMC were cultured in SFM with increasing concentrations of SAP in the absence or presence of 1 μg/ml PTX3 (Fig. 4). In the presence of PTX3, the inhibitory activity of SAP was maintained (Fig. 4). The SAP IC50 for inhibiting fibrocyte differentiation was 0.14 ± 0.05 μg/ml in the absence of PTX3, and 0.10 ± 0.02 μg/ml (mean ± SEM, n = 3, difference not significant by t-test) in the presence of PTX3. There was no significant difference in the dose-response curve Hill coefficient for SAP in the absence (1.6 ± 0.5) or presence of PTX3 (2.2 ± 0.8). These data indicate that PTX3 does not significantly affect the ability of SAP to inhibit fibrocyte differentiation.

### **Fc**γ**RI mediates the effect of PTX3 on murine fibrocyte differentiation**

**Fig 5:**
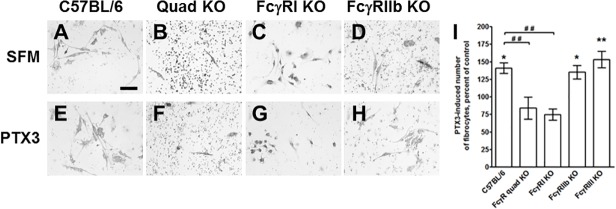
FcγRI KO and quad FcγR KO spleen cells are less sensitive to PTX3 induced fibrocyte differentiation. Spleen cells were cultured for 5 days in the A-D) absence or E-H) presence of PTX3, from A and E) wildtype C57BL/6 mice, B and F) quadruple FcγRI/IIb/III/IV, C and G) FcγRI, and D and H) FcγRIIb knockout mice. After 5 days, cells were air-dried, fixed, and stained, and the number of fibrocytes was counted. Bar is 100 μm. I) For each strain, the count in the presence of PTX3 was normalized to the count in the absence of PTX3. Values are mean ± SEM (n = 3). *p <0.05, **p <0.01 (ANOVA), compared to SFM. ## p<0.01 (ANOVA) compared to C57BL/6.

To determine if PTX3 also promotes murine fibrocyte differentiation, spleen cells from C57BL/6 mice were cultured for 5 days in SFM in the presence or absence of PTX3. Compared to the control, 1 μg/ml PTX3 significantly potentiated murine fibrocyte differentiation (Fig. 5). PTX3 binds to multiple FcγR [34,55]. To determine whether one or more FcγR mediate the potentiating effect of PTX3 on fibrocyte differentiation, we cultured spleen cells from different FcγR knockout mice in the presence or absence of PTX3. PTX3 potentiated fibrocyte differentiation in cultures of spleen cells from FcγRIIb and FcγRIII KO mice (Fig. 5). However, PTX3 did not potentiate fibrocyte differentiation in cultures of spleen cells from FcγRI knockout and FcγRI/IIb/III/IV quadruple knockout mice (Fig. 5). In addition, compared to C57BL/6 spleen cells cultured in the presence of PTX3, there were significantly less fibrocytes in spleen cells from FcγRI knockout and FcγRI/IIb/III/IV quadruple knockout mice cultured with PTX3. By t-test, there were significantly less fibrocytes in cultures of spleen cells from FcγRI and FcγR quad KO when cultured with PTX3, compared to the SFM control, suggesting that in the absence of FcγRI or FcγRVI PTX3 may bind to an additional receptor(s) to generate an inhibitory signal (Fig. 5). These data suggest that FcγRI is required for the potentiating effect of PTX3 on murine fibrocyte differentiation, and that in the absence of FcγR additional unknown receptors may also regulate the effect of PTX3.

### PTX3 binds to human leukocytes and human FcγRI and FcγRIIa on cell lines

Both PTX3 and SAP require FcγRI to regulate fibrocyte differentiation ([32,45,51] and Fig. 5). To determine if the opposing effects of PTX3 and SAP on fibrocyte differentiation were due to these pentraxins binding to distinct or overlapping sites on FcγR, or other pentraxin receptors, we examined the binding of PTX3 and SAP to human leukocytes and to human FcγR expressed on the human-derived cell lines HEK293 and K562.

**Fig 6:**
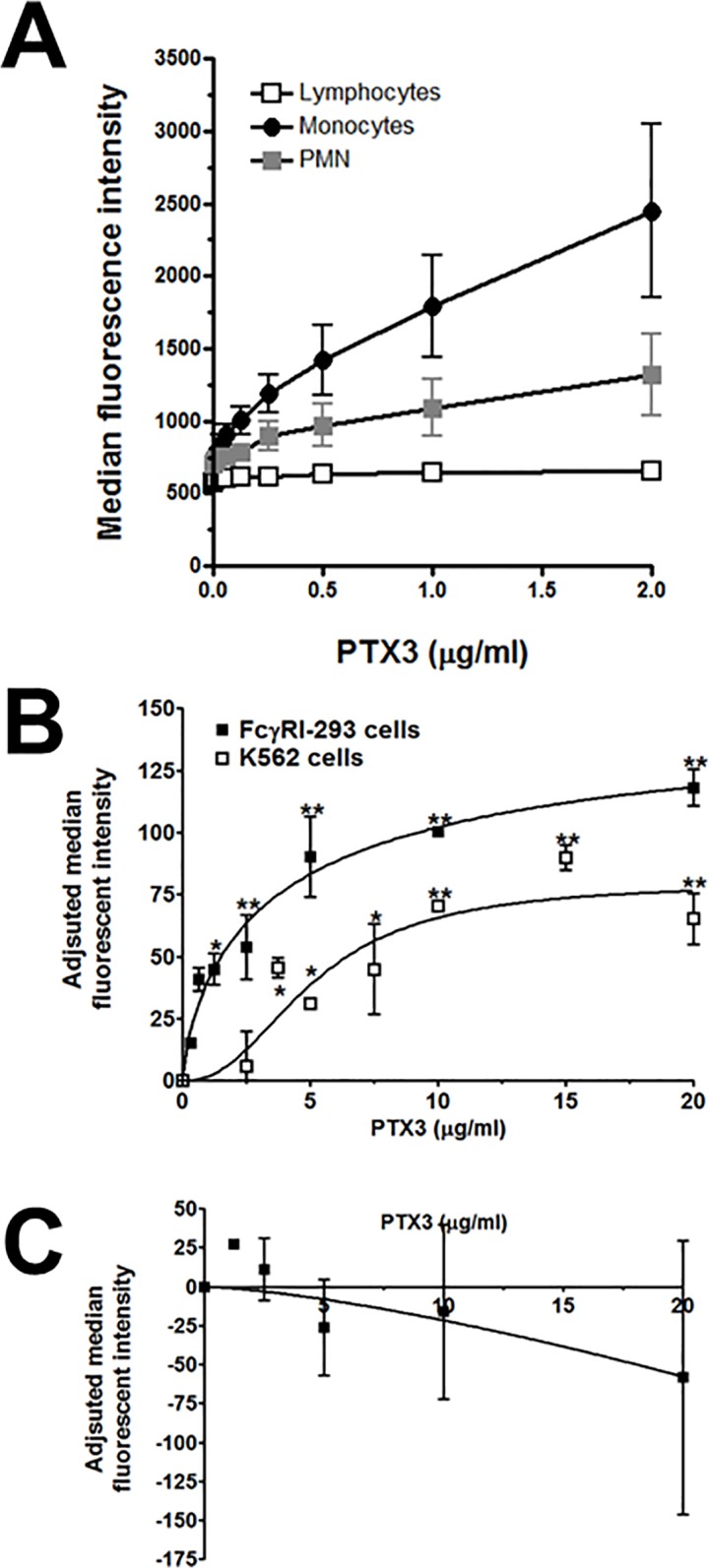
Alexa Fluor 647–labeled PTX3 binds to FcγRI and FcγRIIA. A) PTX3-647 was incubated with isolated leukocytes and then subjected to flow cytometry. Neutrophils, monocytes, and lymphocytes were identified based on forward scatter and side scatter. Curves are fits of the resulting data to models of one or two-site binding with variable Hill coefficient. B) HEK293 cells expressing FcγRI, or K562 cells, which express FcγRIIA and C) HEK293 cells expressing FcγRIIIB were incubated with PTX3-647. The cells were then washed, and the binding of the labeled PTX3 to the cells was measured by flow cytometry. Autofluorescence values were subtracted from the total binding values. Median fluorescence intensity values were normalized to the intensity value of 1 μg/ml concentration. Values are normalized mean ± SEM, n = 3–6. Curves are fits to models of one-site binding with variable Hill coefficient. The absence of error bars indicates that the error was smaller than the plot symbol. *p <0.05, **p <0.01 (1-way ANOVA, Dunnett's test), compared to no PTX3-647 binding.

We determined the binding of Alexa647-labelled PTX3 and SAP to human lymphocytes, monocytes, and neutrophils, as identified by their forward and size scatter characteristics and receptor expression using flow cytometry (Fig. 6A). When PTX3-647 was incubated with leukocytes, we observed minimal binding to the lymphocyte population (Fig. 6A). Because B cells (∼5% of lymphocytes) express FcγRIIB [36,45], and NK cells (∼10% of lymphocytes) express FcγRIIIA [36,45], this suggests that PTX3 does not bind to these receptors under our experimental conditions. However, PTX3-647 did bind to monocytes and neutrophils (Fig. 6A). All monocytes express FcγRI, FcγRIIA, and some monocytes also express FcγRIIIA [6,45]. This indicates that PTX3 could be binding to any or all of the FcγRs on monocytes. Because NK cells express FcγRIIIA, and we did not detect binding of PTX3-647 to NK cells, this suggests that PTX3 binds to FcγRI and/or FcγRIIA on monocytes. Neutrophils express FcγRIIA and FcγRIIIB [6,45]. This then suggests that PTX3 binds to FcγRIIA or possibly FcγRIIIB on neutrophils. PTX3 bound to lymphocytes with a single binding site and a K_D_ of 0.5 ± 1.6 ng/ml. However, both monocytes and neutrophils bound PTX3 with two-site binding characteristics. Monocytes had a high affinity binding site with a K_D_ of 1.3 ± 4.3 ng/ml, and a low affinity binding site with a K_D_ of 3400 ± 6500 ng/ml. Neutrophils had a high affinity binding site of 0.2 ± 2.9 ng/ml, and a low affinity binding site of 2200 ± 6600 ng/ml. By 2-way ANOVA, the differences in both the binding of PTX3 and cell type are significant, p < 0.0001 and p = 0.0296 respectively.

In addition, we assessed the binding of PTX3-647 to K562 cells, which, with respect to FcγR, only express FcγRIIA [54], and HEK293 cells transfected with either human FcγRI or FcγRIIIB (Fig. 6B and C). We observed significant PTX3-647 binding to HEK293 cells expressing FcγRI and PTX3-647 binding to K562 cells (Fig. 6B). We saw no significant PTX3 binding to HEK293 cells expressing FcγRIIIB (Fig. 6C). Together, our data indicate that PTX3 binds to FcγRI and FcγRIIA on human monocytes, neutrophils, and cell lines, but not to FcγRIIB, FcγRIIIA, and FcγRIIIB (Fig. 6).

**Fig 7:**
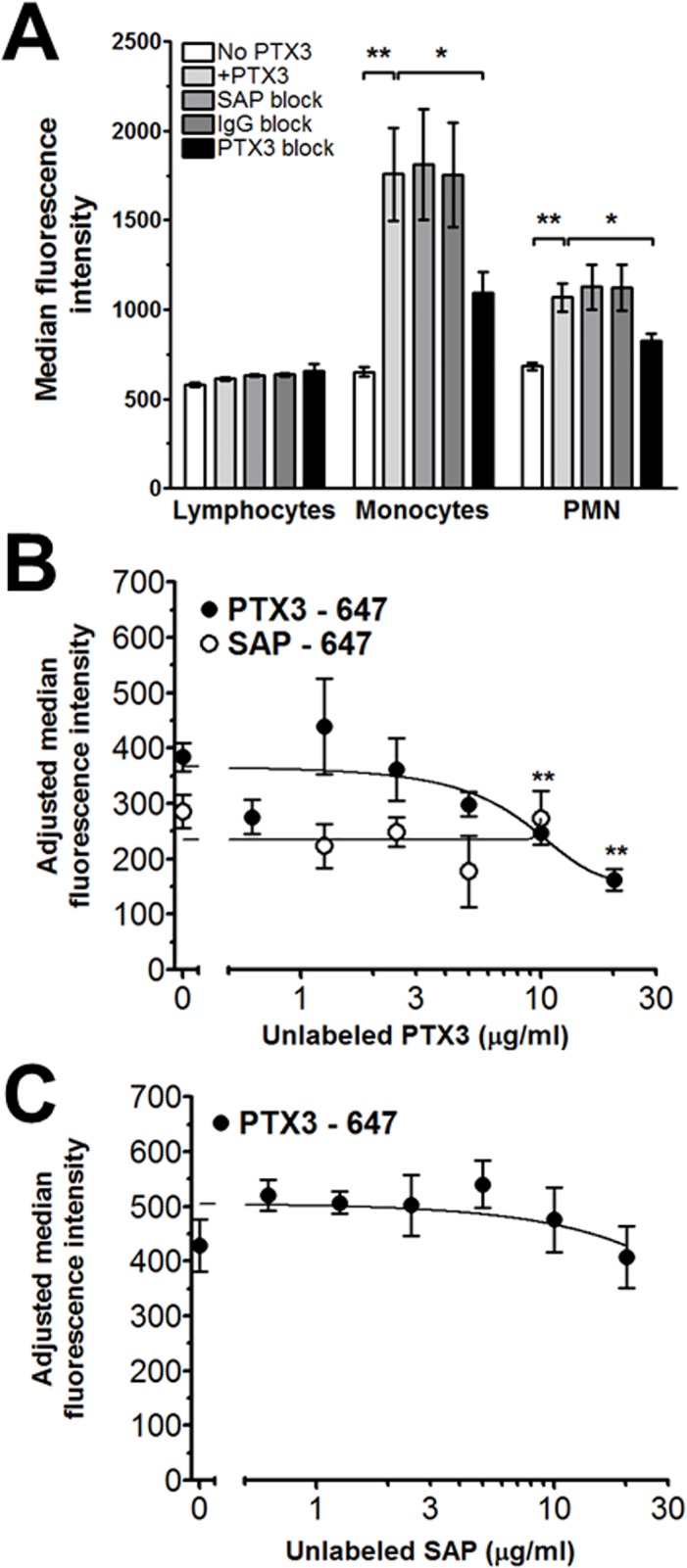
PTX3 and SAP bind to overlapping sites on leukocytes. A) Human Leukocytes were incubated with 1 μg/ml unlabeled PTX3, SAP, or human IgG Fc for 30 minutes at 4°C. Cells were then incubated with 1 μg/ml labelled PTX3, for an additional 30 minutes at 4°C. The cells were then washed, and the binding of the labeled PTX3 to the cells was measured by flow cytometry. Values are mean ± SEM, n = 5. * p <0.05, ** p <0.01 (ANOVA), compared to cells incubated with PTX3-647. HEK293 cells expressing FcγRI were incubated with B) PTX3-647 or SAP-647 in the presence of unlabeled PTX3, or C) PTX3-647 and unlabeled SAP. The cells were then washed, and the binding of the labeled pentraxin to the cells was measured by flow cytometry. Autofluorescence values were subtracted from the total binding values. Values are normalized mean ± SEM, n = 3. Lines are fits to models of one-site binding with variable Hill coefficient or linear regression. ** p <0.01 (t-test), compared to cells incubated with PTX3-647 alone.

To determine if PTX3, SAP, and IgG bind to similar or distinct sites on human leukocytes and on HEK293 cells expressing human FcγRI, we pre-incubated cells with unlabeled PTX3, SAP, or human IgG1 Fc, and then Alexa 647-labelled PTX3. We observed that on monocytes, PTX3-647 binding was inhibited by unlabeled PTX3, but not SAP or IgG Fc (Fig. 7A). On HEK293 cells transfected with human FcγRI, we observed that unlabeled PTX3 could inhibit PTX3-647 binding (Fig. 7B). In similar assays, unlabeled SAP inhibits SAP binding to FcγRI expressing macrophages [22], and we observed that unlabeled SAP could not inhibit PTX3-647 binding to FcγRI (Fig. 7C). In addition, unlabeled PTX3 could not inhibit SAP-647 binding to FcγRI (Fig. 7B). These data suggest that PTX3 and SAP bind to distinct sites on FcγRI, and that PTX3 may also binds to sites that are distinct from IgG.

### Expression of PTX3 in lung cells, murine models of fibrosis, and pulmonary fibrosis patients

**Fig 8:**
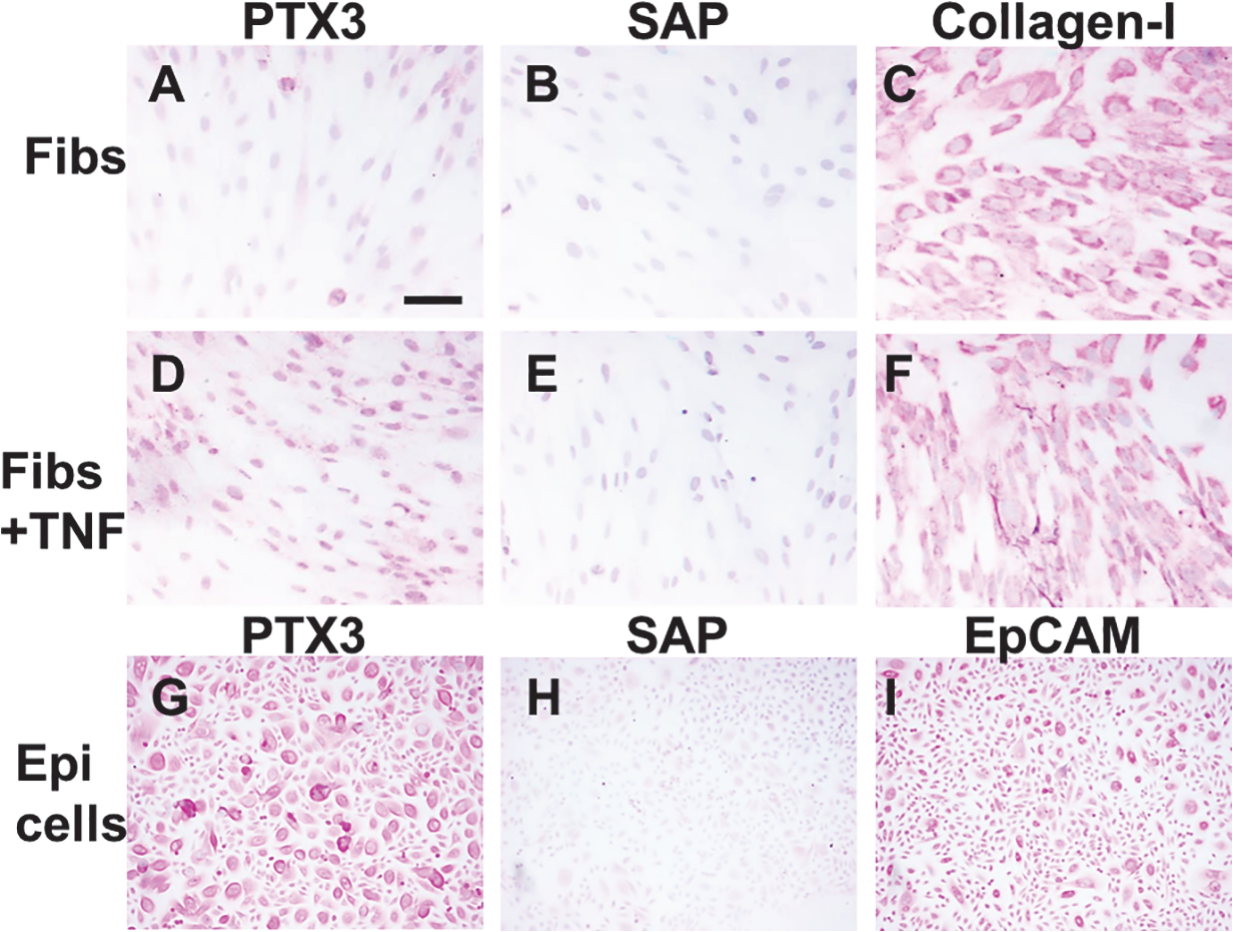
Human lung fibroblasts and epithelial cells express PTX3. Human A-C) lung fibroblasts, D-F) lung fibroblasts incubated with 50 ng/ml TNF-α, or G-I) lung epithelial cells were cultured for 2 days in 8-well glass slides. Cells were then air-dried, fixed, and labeled with A, D, and G) anti-PTX3 antibodies, B, E, and H) anti-SAP antibodies, C and F) anti-collagen I antibodies, or I) anti-EpCAM antibodies. Positive staining was identified by red staining, and nuclei are counterstained blue. Bar is 100 μm.

Fibrocytes are found in pulmonary fibrosis lesions, and PTX3 accumulation is associated with fibrotic lesions in asthma and myocardial infarction [3,58–60]. In addition, PTX3 is expressed by dermal fibroblasts and renal epithelial cells and upregulated by TNF-α [61–63]. We observed that human lung fibroblasts and bronchial epithelial cells also produce PTX3, and as with the dermal fibroblasts, TNF-α upregulated PTX3 in lung fibroblasts (Fig. 8). We did not detect the expression of SAP by human lung fibroblasts and epithelial cells (Fig. 8). These data indicate that the local production of PTX3 by fibroblasts and epithelial cells may augment the differentiation of monocytes into fibrocytes in fibrotic lesions.

**Fig 9:**
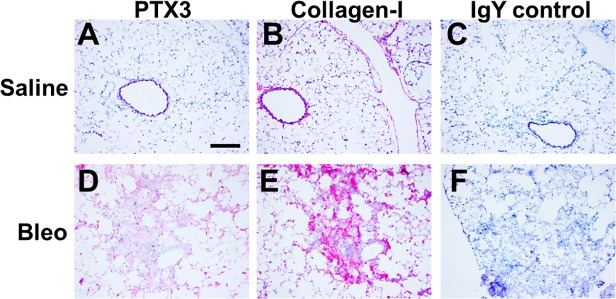
Distribution of PTX3 in mouse lungs following bleomycin aspiration. Cryosections of mouse lungs 21 days after A-C) saline or D-F) bleomycin aspiration were incubated with antibodies against A and D) PTX3, B and E) collagen-I, or C and F) control rabbit antibodies Sections were counterstained with hematoxylin. Bar is 0.2 mm. Images are representative of 3 independent experiments.

To determine if PTX3 was also upregulated in pulmonary fibrosis, we stained lung tissue from mice that aspirated bleomycin or saline. At 21 days after bleomycin aspiration, compared to mice that received saline, mice that aspirated bleomycin had more PTX3 staining, and this was associated with areas of increased collagen-I staining (Fig. 9). These data suggest that bleomycin may lead to the increased local production of PTX3 and this may promote increased fibrocyte differentiation.

**Fig 10:**
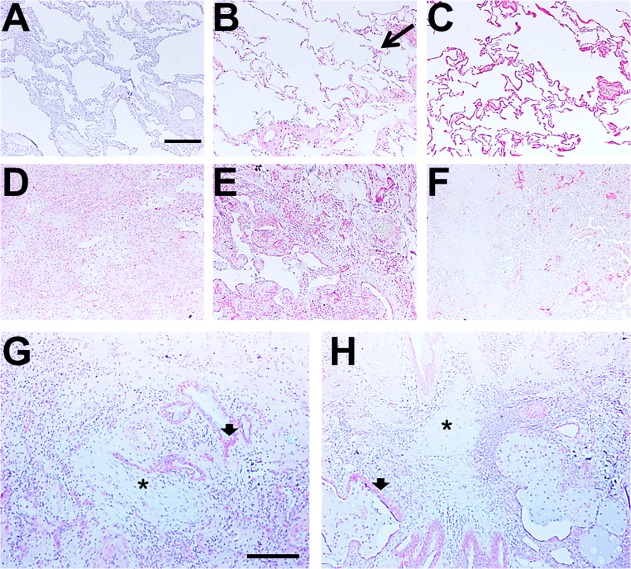
The distribution of PTX3 and SAP in COPD and pulmonary fibrosis lung tissue. Human lung tissue from A-C) COPD patients or D-H) pulmonary fibrosis patients was stained with A) control rabbit antibodies, B, D, E, G and H) anti-PTX3 or C and F) anti-SAP antibodies. Tissues were counterstained with hematoxylin. Positive staining is identified by red color, and nuclei are counterstained blue. Bar is 0.2 mm. Long arrow indicate PTX3 staining in an alveolar septum, short arrow indicates PTX3 staining in lung epithelium, and asterisks indicate fibroblastic foci.

**Fig 11:**
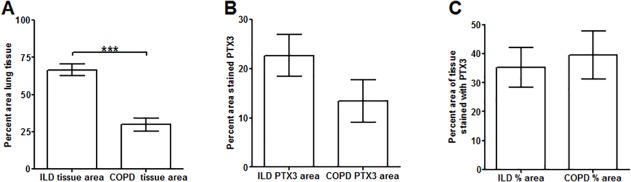
Quantification of PTX3 staining in COPD and pulmonary fibrosis lung tissue. A) The percentage of total area of image containing lung tissue, B) the percentage area of lung tissue stained by PTX3 antibodies, and C) the percentage of lung tissue stained by PTX3 antibodies as a percentage of the total area of the lung. Values are mean ± SEM, n = 7–9 patients per group. *** indicates p < 0.0001 (t-test).

To determine if PTX3 is associated with human lung fibrosis, we examined the distribution of PTX3 in lung tissue from chronic obstructive pulmonary disease (COPD) patients with relatively normal lungs (> 80% FVC) and idiopathic pulmonary fibrosis (IPF) patients (<50% FVC) (Table 1) (Fig. 10). Lung tissue from COPD patients showed a widespread distribution of PTX3, especially in the alveolar septa (Fig. 10B). In the lung tissue from pulmonary fibrosis patients, PTX3 was distributed throughout the tissue, including the lung epithelium, alveolar leukocytes, and fibrotic areas, but PTX3 distribution was reduced within fibroblastic foci (Fig. 10D-H). As with PTX3, there was a widespread distribution of SAP throughout the lung tissue from COPD patients (Fig. 10C). However, in fibrotic lesions from pulmonary fibrosis patients, SAP was restricted to areas adjacent to vessels, and apparently absent from the fibrotic areas (Fig. 10F). However, when we quantified the staining levels for the expression of PTX3 to the total lung tissue area, there were no significant differences between COPD and IPF patients (Fig. 11). These data suggest that the expression of PTX3 by the lung tissue is not due to increased expression of PTX3 by cells, but that the increased number of cells leads to increased PTX3 expression. These data also indicate that PTX3 and SAP are present throughout lung tissue with a relatively normal architecture, while PTX3 and a relatively small amount of SAP are present in fibrotic lesions.

**Table 1:** Clinical data.

Group	FVC (mean±SD)	Gender	Age in years (mean±SD)	Clinical Details
**ILD <50%**	34.25 ± 7.47	5 male; 3 female	49.25 ± 12.41	n = 5 UIP; n = 1 NSIP; n = 2 fibrosis
**COPD >80%**	89.29 ± 7.38	2 male; 5 female	74.00 ± 9.09	n = 2 COPD; n = 4 Emphysema with carcinoma; n = 1 emphysema
**t-test (Mann-Whitney)**	p = 0.0003	ns	p = 0.0006	

Clinical data from the National Heart Lung and Blood Institute-sponsored Lung Tissue Research Consortium (LTRC) sections used in Figs. 10 and 11. Pulmonary function test—Forced vital capacity (FVC). Clinical diagnosis: Chronic obstructive pulmonary disease (COPD); Fibrosis – indicates uncharacterized interstitial lung disease (ILD); Usual interstitial pneumonia (UIP); Non-specific interstitial pneumonia (NSIP).

## Discussion

We found that PTX3 promotes fibrocyte differentiation by a FcγRI dependent mechanism. However, the fibrocyte-inhibitory activity of SAP is dominant over PTX3. In fibrotic lung tissue, we found that the distribution of PTX3 was widespread, and present in alveolar macrophages, lung epithelial cells, and fibroblasts. However, SAP had a restricted distribution and was apparently absent from the fibrotic areas. These data suggest that the relative levels of SAP and PTX3 present at sites of fibrosis may have a significant effect on the ability of monocytes to differentiate into fibrocytes.

Fibrocytes have been detected in human pathological conditions including pulmonary fibrosis [3,10,64–68], keloid scars [69], asthma [64], chronic kidney disease [11], and nephrogenic systemic fibrosis [70]. Fibrocytes are also present in the fibrotic lesions in animal models of pulmonary fibrosis [7,9,65,71–77], liver fibrosis [9] and renal fibrosis [76,78]. In addition to contributing to the mass of fibrotic lesions, fibrocytes promote angiogenesis [79,80], which can then promote the growth of the lesion, and secrete TGF-β [81], which activates resident fibroblasts. Therefore, in situations where PTX3 is abundant and therefore available to promote fibrocyte differentiation, this may also regulate angiogenesis. Finally, the injection of mature fibrocytes into mice potentiate lung fibrosis, suggesting that the increased recruitment of monocyte-derived fibrocytes may potentiate an ongoing fibrotic response [72]. Whether the main role of fibrocytes is to directly drive fibrosis, through the production of extracellular matrix proteins, or to act in a paracrine manner to activate stromal cells (fibroblasts) to produce more extracellular matrix proteins, or regulate angiogenesis, is still unclear [3,80,82].

Compared to human lung tissue, the lungs of saline-treated mice expressed little PTX3. Whether this is a difference in PTX3 expression between human and murine lungs, or due to elevated PTX3 expression even in COPD patients compared to healthy controls, is unclear. In both LPS and ventilator-induced lung injury models in rats, PTX3 was detectable in sham/control lung samples [83,84], but in a smoke inhalation model in mice PTX3 was not expressed by lung cells in control animals [85]. In humans, some studies have detected PTX3 in normal lung tissue [86], whereas in samples from invasive pulmonary aspergillosis, only alveolar macrophages and not the lung tissue expressed PTX3 [87]. These data suggest that not only the severity and length of the insult may regulate the expression of PTX3 by lung cells, but that the expression of PTX3 in lung cells may also vary between species [88].

Although the concentration of PTX3 found to promote fibrocyte differentiation is higher than detected in circulating plasma, the concentration used is 10 fold lower than used in most other *in vitro* systems [89–92]. In addition, the plasma concentration of PTX3 is unlikely to represent the concentration present in the tissues, especially during an immune response. Although it is difficult to determine the actual concentration of PTX3 in tissues, using data from cells cultured *in vitro* may provide information to permit an approximation for the levels of PTX3 in tissues. Many cells present at sites of inflammation or fibrosis, including endothelial cells, fibroblasts, neutrophils, and macrophages secrete PTX3, with levels ranging from 20–100 ng per 10^6^ cells [17,93–95]. In murine models of lung inflammation and fibrosis, there can be 10 x 10^6^ leukocytes present in the vascular, interstitial, and alveolar compartments of the lung [96,97]. As a model for lung fibrosis, bleomycin instillation into the lung generates patchy inflammation and fibrosis with typically 10–20% of the lung tissue affected [98,99]. Therefore, if we assume that the majority of the infiltrating cells associate with sites of tissue injury [99], this would suggest that approximately 10 x 10^6^ cells would be confined to specific areas of lung tissue. As 10^6^ cells secrete 50–100 ng PTX3, and 10^6^ cells occupy approximately 100 mm^3^ (100 mm^3^ is 100 μl) this suggests that the local concentration of PTX3 within tissues could reach 1 μg/ml, and be even higher within the lung interstitium.

Surface plasmon resonance experiments show that SAP and CRP, bind to all of the human FcγR, whereas PTX3 only binds to human FcγRIII and weakly to human FcγRIIA [22,34]. However, we observed that PTX3 binds to FcγRI and FcγRIIA on leukocytes, K562 cells, and transfected HEK293 cells. This inconsistency with the previously published data may be explained by the differences in the glycosylation state of the receptors and/or the lack of some intracellular signaling components that promote receptor binding [37]. As FcγRI and FcγRIIIA lack an intrinsic motif that binds to intracellular signaling components, they interact with the intracellular protein FcRγ. The absence of FcRγ reduces the affinity of FcγRI and FcγRIIIA for IgG in humans [37,100]. This can potentially alter PTX3 binding to FcγRI and FcγRIIA. Together, this suggests that the PTX3 affinity for FcγRs is dependent on the modification of these FcγRs and the interactions they make before binding PTX3.

There are several possible explanations for the observation that SAP appears to signal through FcγRI on monocytes to inhibit fibrocyte differentiation, while PTX3 appears to signal through the same receptor on the same cells to promote fibrocyte differentiation [32]. At first glance, it would appear that SAP might be an agonist, and PTX3 an inverse agonist (or vice versa) of FcγRI, but the observation that in serum-free medium, mouse cells lacking FcγRI show only slightly reduced levels of fibrocyte differentiation, suggests that there is little constitutive signaling from FcγRI with respect to promoting or inhibiting fibrocyte differentiation [32,51].

In humans, the main activating FcγR on monocytes are FcγRI, FcγRIIA, and FcγRIIIA, whereas in mice the main activating FcγR on monocytes are FcγRI, FcγRIII, and FcγRIV [38,101]. Human FcγRI is orthologous to mouse FcγRI, human FcγRIIA is most closely related to mouse FcγRIII, and human FcγRIIIA is most closely related to mouse FcγRIV [38,102]. We found that the FcγRI knockout and FcγRI/IIb/III/IV quadruple knockout mice spleen cells were insensitive to PTX3. We did not have access to the single FcγRIV knockout mouse spleen cells, but the PTX3 effect on human CD14+CD16- (FcγRIIIA-) monocytes and the lack of PTX3 binding to FcγRIII on leukocytes and HEK293 cells, suggests that human FcγRIIIA or murine FcγRIV are not essential for PTX3 activity. Combined with our previous observation that SAP inhibits human and murine fibrocyte differentiation mainly through FcγRI, these results suggest that FcγRI plays a major role in regulating fibrocyte differentiation [32,41].

Fc receptors can differentially activate signaling cascades depending on the affinity or avidity of their ligands [103,104]. Therefore, the differential effect of SAP and PTX3 on fibrocyte differentiation may be due to the pentameric structure of SAP, compared to the decameric structure of PTX3, or the affinity of these pentraxins for FcγR [5,34,105,106]. FcγRI may thus exhibit functional selectivity/biased agonism, with SAP preferentially activating one downstream pathway, and PTX3 activating a different pathway [107–109]. We have previously observed that although SAP and aggregated IgG both inhibit fibrocyte differentiation, aggregated IgG inhibits fibrocyte differentiation through a pathway involving Syk (a non-receptor cytoplasmic tyrosine kinase) but that SAP appears to signal through a Syk-independent pathway [41]. A second possibility is that a different receptor modulates the signal from FcγRI. Mouse cells lacking FcγRI, lacking the common FcRγ chain, or lacking all four FcγR receptors still show inhibition of fibrocyte differentiation by SAP, albeit with an increased IC50 [32]. This then suggests that a different receptor binds SAP, and this receptor could thus modulate the signal from FcγRI so that SAP binding to FcγRI and the unknown receptor would inhibit fibrocyte differentiation, and PTX3, binding to FcγRI and not binding to the unknown receptor, or additionally binding to a third receptor, would increase fibrocyte differentiation. SAP and CRP, but not PTX3, also bind to the IgA receptor (CD89) [110]. However, we have previously shown that IgA does not regulate human fibrocyte differentiation [41], and in mice the IgA receptor is absent [111]. Although mice do not have CD89, they express Fcα/μR, a dual IgA/IgM receptor, which is independent of FcRγ [112], however we have previously shown that IgM does not regulate human fibrocyte differentiation [41]. Therefore, additional unknown receptor(s) may well be involved in the regulation of fibrocyte differentiation by pentraxins. Since fibrocytes play a key role in fibrosis, and FcγRI plays a key role in regulating fibrocyte differentiation, our results on the effects of SAP and PTX3 indicate that FcγRI signaling represents an interesting new therapeutic target.
